# Cross-Category Tea Polyphenols Evaluation Model Based on Feature Fusion of Electronic Nose and Hyperspectral Imagery

**DOI:** 10.3390/s20010050

**Published:** 2019-12-20

**Authors:** Baohua Yang, Lin Qi, Mengxuan Wang, Saddam Hussain, Huabin Wang, Bing Wang, Jingming Ning

**Affiliations:** 1 School of Information and Computer, Anhui Agricultural University, Hefei 230036, China; 2 New Rural Research Institute, Anhui Agricultural University, Hefei 230036, China; 3 School of Electrical and Information Engineering, Anhui University of Technology, Ma’anshan 243032, China; 4 State Key Laboratory of Tea Plant Biology and Utilization, Anhui Agricultural University, Hefei 230036, China

**Keywords:** tea polyphenols, feature fusion, electronic nose, hyperspectral

## Abstract

Tea polyphenols are important ingredients for evaluating tea quality. The rapid development of sensors provides an efficient method for nondestructive detection of tea polyphenols. Previous studies have shown that features obtained from single or multiple sensors yield better results in detecting interior tea quality. However, due to their lack of external features, it is difficult to meet the general evaluation model for the quality of the interior and exterior of tea. In addition, some features do not fully reflect the sensor signals of tea for several categories. Therefore, a feature fusion method based on time and frequency domains from electronic nose (E-nose) and hyperspectral imagery (HSI) is proposed to estimate the polyphenol content of tea for cross-category evaluation. The random forest and the gradient boosting decision tree (GBDT) are used to evaluate the feature importance to obtain the optimized features. Three models based on different features for cross-category tea (black tea, green tea, and yellow tea) were compared, including grid support vector regression (Grid-SVR), random forest (RF), and extreme gradient boosting (XGBoost). The results show that the accuracy of fusion features based on the time and frequency domain from the electronic nose and hyperspectral image system is higher than that of the features from single sensor. Whether based on all original features or optimized features, the performance of XGBoost is the best among the three regression algorithms (*R^2^* = 0.998, RMSE = 0.434). Results indicate that the proposed method in this study can improve the estimation accuracy of tea polyphenol content for cross-category evaluation, which provides a technical basis for predicting other components of tea.

## 1. Background

Tea polyphenols (TP), as the main biological active ingredient in tea, affects the aroma of tea and the volatility of flavor compounds [1]. Different tea varieties have different polyphenol contents, which is one of the key indicators for assessing tea quality [2] and which affects tea quality control. In addition, tea polyphenols have attracted the attention of scholars at home and abroad because of their various pharmacological effects [3], which makes the quantitative extraction and detection of tea polyphenol content especially important [4]. Therefore, the establishment of an efficient tea polyphenol detection model is of great significance for tea quality improvement and function expansion.

In recent years, many chemical analysis methods have been used to determine the total polyphenol content in tea, such as gas chromatography (GLC), capillary electrophoresis, and high-performance liquid chromatography (HPLC) [5,6,7], which have achieved good results. However, they still have some disadvantages, such as low detection efficiency, high destructiveness, and high detection cost, which cannot meet the requirements of real-time detection of tea quality. Therefore, it is important to find a rapid and nondestructive detection method. In the last ten years, with the development of spectroscopic instruments and data processing technologies, there have been many studies on the detection of phenolic components in tea. However, they mainly focus on the general models of green tea, black tea, and oolong tea to detect tea polyphenols [8,9,10,11,12,13,14]. At present, there is still a lack of a general model to evaluate the quality cross-category tea parameters. Therefore, the nondestructive detection of polyphenols from cross-category teas still faces great challenges.

With the rapid development of sensors, the wide application of electronic nose (E-nose), electronic tongue, and near-infrared technology [15,16,17,18,19,20,21,22] has made tea quality estimation easier. Especially, electronic nose technology has the convenience and objectivity of detecting food taste, which has been successfully applied to many aspects of tea research by simulating the human olfactory system, including in the tea fermentation process [23,24], tea classification [25,26,27,28], tea storage [29], and tea components [30]. However, the function of a single sensor always has certain limitations [31]. Therefore, it is necessary to study the combination of different technologies to capture more comprehensive information. Many studies report that combining multiple sensor signals can improve the results of tea quality estimates. For example, the combination of electronic nose and capillary electrophoresis [32], the combination of electronic nose and visible/near infrared spectroscopy [33], and the combination of electronic nose and electronic tongue technologies [34,35,36]. All of the above studies have achieved good results, regardless of whether they used a single sensor or a multisensor, which obtains signal features or functional group features that can reflect changes in the internal composition of the tea. However, the lack of spatial information limits the in-depth study of tea polyphenols. Actually, different varieties of tea might have different spatial characteristics, even if they come from the same category, not to mention that different categories of tea certainly have obviously different spatial characteristics. Therefore, it is necessary to study the spatial features of tea to make up for the lack of information. To date, hyperspectral imaging technology has been used to improve the evaluation of tea components, including polyphenols [37,38,39], amino acid [40], and catechins [41], due to the advantages of simultaneous acquisition of spatial image information and spectral information of the analyte. However, it remains unclear whether it is possible to improve the estimation model of the polyphenol content of cross-category tea based on the fusion features of different sensors.

In fact, how to effectively fuse multiple features based on E-nose and hyperspectral imagery (HSI) still faces many problems. On the one hand, there is the question of how to extract more meaningful features. On the other hand, there is the question of how to select more representative features. Previous studies have shown that multisource information fusion can more effectively detect the composition of tea [33]. Moreover, the time domain and frequency domain features are more effective in extracting internal quality from the sensor signal array [42]. However, there are still some obstacles to the acquisition of features of cross-category tea. The wide application of wavelet transforms [43,44] provides a new idea for the analysis of tea quality, since this method can extract the time domain and frequency domain features of the signal [45].

Therefore, this study focused on the feasibility of fusion features from multisource sensors, including E-nose and HSI, to estimate the polyphenol content of cross-category tea. To make full use of time domain features and frequency domain features, support vector regression (SVR) [46], random forest (RF) [47], and extreme gradient boosting (XGBoost) [48] are used to construct an estimation model to improve the accuracy of tea polyphenol content of cross-category.

The purpose of this study is to: (1) extract time domain features and frequency domain features from the electronic nose and hyperspectral systems, respectively; (2) fuse time domain features and frequency domain features based on E-nose and HSI to improve estimation models of polyphenol content for cross-category tea; and (3) compare and evaluate polyphenol content estimation models for cross-category tea based on three different regression methods.

## 2. Data and Methods

### 2.1. Sample Collection

A total of 110 samples of tea (three categories: yellow tea, black tea, and green tea) were collected from different provinces of China, which were kept in a closed jar and stored in a refrigerator at about 4 °C before test. In order to obtain a wide range of tea polyphenols, different geographical origins of tea samples were collected for the same variety of tea for experimentation.

For the same category of tea, different varieties (geographical origins) of tea samples were collected for experimentation. For example, three brands were collected for black tea, including Zhengshan Xiaozhong tea, Qimen Black tea (Anhui Huangshan and Anhui Qimen, China), and Jinjunmei tea. Therefore, 10 varieties were obtained, as shown in Table 1, 10–15 samples were selected for each variety, and 110 samples were used as research objects.

The tea polyphenol content was directly titrated by potassium permanganate. The tea polyphenol content was calculated according to the Equation (1):

(1) $$X=\frac{(A-B)\times \omega \times 0.00582/0.318}{m\times V_{1}/V_{2}}$$

where X represents the content (%) of tea polyphenols; A and B represent the number of milliliters of potassium permanganate consumed by the sample and the number of milliliters of potassium permanganate consumed in the blank, respectively; and *ω* represents the concentration of potassium permanganate (%), m represents the mass (g) of the sample, and V_1_ and V_2_ represent the volume (mL) of the test solution and the test solution for measurement, respectively.

### 2.2. Sample Sampling

#### 2.2.1. HSI Sampling

The hyperspectral image acquisition system used in this experiment included a near-infrared spectrograph (Imspector V17E, Spectral Imaging Ltd., Oulu, Finland), which covers the spectral range of 900–1700 nm, and a charge couple device (CCD)-based digital video camera (IPX-2M30, Imperx Inc., Boca Raton, FL, USA), two 150W halogen lamps (3900, Illumination Technologies Inc., New York, NY, USA), one data acquisition black box, reflective linear tube, electronically controlled displacement platform (MTS120, Beijing Optical Instrument Factory, Beijing, China), and the image acquisition and analysis software (Spectral Image Software, Isuzu Optics Corp., Zhubei, Taiwan). The four tungsten halogen lamps of the reflected light source are evenly distributed on the ring bracket in the dark box, and the light source is irradiated in a direction of 45° with respect to the vertical direction.

The parameters when collecting hyperspectral images are set as follows: exposure time of 20 ms, electronic control stage moving speed of 8 mm·s^−1^, image resolution of 636 × 815 pixels; spectral resolution of 5 nm, spectral range of 908–1700 nm, spectral sampling interval of 2 nm. Here, 20 g ± 0.5 g from each variety tea sample was evenly spread in a Φ 9 × 1 cm culture dish, and was placed on an electronically controlled stage in a black box to collect hyperspectral images of tea, which were black and white calibrated to remove noise interference and other light source interference.

#### 2.2.2. E-Nose Sampling

The electronic nose (PEN3, Win Muster Air-sense Analytics Inc., Schwerin, Germany) used to collect the scent fingerprint of tea has many functions, including automatic adjustment, automatic calibration, and automatic enrichment, and consists of three units, including a gas sensor array, a signal preprocessing unit, and a pattern recognition unit. The gas sensor array is composed of 10 metal oxide sensors (MOS) (W1C, W5S, W3C, W6S, W5C, W1S, W1W, W2S, W2W, and W3S), which are defined as f0–f9 and are sensitive to different types of volatiles. The characteristics of each sensor are shown in Table 2. 

Zero gas was pumped into the cleaning channel to reset the sensors before sampling, and 5 g of each tea sample was placed in a 100 mL glass beaker, sealed and placed for 30 min, and the gas in the headspace bottle was equilibrated and tested. The parameters of the electronic nose were set as follows: sampling interval of 1 s, sensor cleaning of 60 s, sensor return time of 10 s, sampling time of 75 s, and injection flow rate of 600 mL/min.

### 2.3. Feature Extraction

#### 2.3.1. Feature Extraction from E-Nose System

Feature extraction is used to extract the useful information of a sensor signal by a certain means, so that the discrimination between different types of signals is highlighted and maximized, which is an important part of the model establishment process. The time domain feature mainly measures the change of the signal with time. By comparing the waveform shape of the electrical signal, small differences in odor can be obtained for different samples.

In order to achieve quantitative evaluation of multiple features, parameters including variance, integrals, steady state average, mean differential value, skewness, and kurtosis were selected as time domain features, as shown in Table 3. Variance describes the degree of data dispersion acquired by different sensors, the integral value reflects the total response of the sensor to the gas, the steady state average reflects the characteristic information of the sample, the average differential value reflects the average speed of the sensor’s response to the gas, and skewness and kurtosis reflect the distribution of signals [49,50,51,52].

Here, $c_{i}$ represents the response of the sensor to the second of the sample; $\bar{c}$ is the average of the response of the signal; N is the acquisition time of a sample, where N=75; Δt is the time interval between two adjacent acquisition points (Δt = 1 s); $t_{0}$ is the time corresponding to when the steady state is ready to be reached.

The frequency domain feature mainly depicts the frequency distribution of the signal. Signal-based frequency domain transformation generally analyzes the energy and entropy values of signals at various frequencies. Wavelet transform is one of the potential technologies for frequency domain information extraction. It can decompose signals into subsignals of different frequencies and effectively express different features. Therefore, the maximum energy and mean of the wavelet transform coefficients are used to represent the main characteristics and overall level of the sensor signal.

Here, $X_{i}$ is the wavelet coefficient of CWT processed by $2^{i}$ layers, i represents the different scale factors (i=1,2,3…10), the wavelet coefficient energy is the square of the wavelet coefficient at each scale, $E_{i}$ is the wavelet coefficient energy, and S is the sum of the wavelet coefficient energy. $W_{M}$ and $W_{A}$ represent the maximum and mean of the sum of the energy of the wavelet coefficients [53]:

(2) $$E_{i}=X_{i}^{2}$$

(3) $$S_{j}=\sum_{i=1}^{n}E_{ij}\;(j=1,2,\ldots 10\;\mathrm{for}\;E\text{-}\mathrm{nose})$$

(4) $$W_{M}=max\left|S_{1},S_{2},\ldots S_{j}\ldots S_{n}\right|(j=1,2,\ldots 10\;\mathrm{for}\;E\text{-}\mathrm{nose})$$

(5) $$W_{A}=\frac{1}{n}\sum_{j=1}^{n}S_{j}(j=1,2,\ldots 10\;\mathrm{for}\;E\text{-}\mathrm{nose})$$

#### 2.3.2. Feature Extraction from HSI

In image processing, the time domain is the scanning of signals at different times, the processing object is the image itself, and the frequency domain is a coordinate system used to describe the frequency characteristics of a signal. In the frequency domain, the information of the image appears as a combination of different frequency components. Wavelet transform is a time frequency analysis method based on Fourier transform, which can simultaneously represent features including time domain and frequency domain.

Therefore, the wavelet transform is used to obtain the energy ($W_{E}$) and entropy ($W_{EN}$) characteristics of sub-images of different frequencies from the hyperspectral image. In this study, the Daubechies wavelet is used to decompose the hyperspectral image in two layers, $W_{E}$ and $W_{EN}$, for the image according to Equations (6) and (7) [54]:

(6) $$W_{E}=\frac{1}{MN}{\sum_{m=1}^{M}\sum_{n=1}^{N}\left|I_{mn}^{\Lambda }\right|}^{2}$$

(7) $$W_{EN}=-\frac{1}{MN}\sum_{m=1}^{M}\sum_{n-1}^{N}I_{mn}^{\Lambda }(\log2(I_{mn}^{\Lambda }))$$

where M×N represents the image, (x,y) is a pixel, $I_{mn}^{\Lambda }$ is the wavelet coefficient, and Λ=|LH,HL,HH|.

### 2.4. Methodology

#### 2.4.1. Normalized Processing

To eliminate the differences between different features to achieve the comparability of multiple indicators, the data needs to be normalized; that is, a dimensionless processing method is required that can reduce the calculation amount and training time. The sensor characteristic data is mapped to 0–1 by the normalization method, and the calculation formula is as shown in the Formula (8).

(8) $$V^{\prime }=\frac{V-V_{\min}}{V_{\max}-V_{\min}}$$

where $V^{\prime }$ and V represent the normalized value and original value, $V_{\max}$ and $V_{\min}$ represent the maximum and the minimum value of the original data.

#### 2.4.2. Support Vector Regression

Support vector regression (SVR) is a linear regression through dimensional transformation. The value of SVR parameters (penalty parameters and kernel parameters) has a great influence on the evaluation performance of SVR. To improve the accuracy of the model, the grid algorithm is used to optimize the selection of SVR parameters [46]. Therefore, the SVR hyperparameter range is shown in Table 4.

#### 2.4.3. Random Forest

Random forest (RF) is an integrated learning method based on bagging, which further improves the level of generalization for the model by selecting random variable sets and random samples from the calibration data set [47], to avoid overfitting caused by excessive eigenvalues and weakening of the influence of outliers on the model. In this study, RF is used to construct the prediction model, and the hyperparameter range of RF is shown in Table 5.

#### 2.4.4. Extreme Gradient Boosting

Extreme gradient boosting (XGBoost) [48] is an integrated algorithm based on lifting trees., which uses the gradient descent architecture to enhance the integrated tree approach of weak learners (typically classification and regression tree). The hyperparameters of the model are optimized with grid search, as shown in Table 6, which is used to build the best model.

#### 2.4.5. Feature Importance Assessment Method

To reduce the number of features and improve the accuracy of the model, feature selection based on the importance of the feature can eliminate irrelevant or redundant features. Therefore, feature selection based on the XGBoost method and correlation coefficient analysis methods are used to detect and eliminate useless features.

In the training process of the XGBoost model, the criterion for dividing each node of the tree is implemented to achieve the optimal features, which indicates the importance of this feature in dividing decision tree nodes. The importance of a feature is the sum of the occurrences of it in all trees. The larger the value, the more important this feature is. Correlation analysis can measure the closeness of the correlation between two characteristic factors [55]. Pearson’s correlation coefficient was used to analyze the correlation between tea polyphenols and the time domain and frequency domain features from HSI. The correlation coefficient value ranges from −1 to 1, and the larger the absolute value, the higher the correlation.

To ensure the representativeness of tea polyphenol content, the data set was divided into a calibration set and validation set according to different varieties of tea. The ratio was about 7:3, and the results are shown in Table 7. The content of tea polyphenols ranged from 10.65% to 29.65% in all samples. The data distribution trend of the calibration set was also consistent with the validation set, indicating that the data distribution of the two data sets was reasonable.

To further verify the universality of the model, the coefficient of determination (*R^2^)*, adjusted determination coefficient (*adjusted_R^2^*) as shown in Formula (9), and the root mean squared error (RMSE) were used as indicators to interpret and quantify the model [56,57].

(9) $$adjusted\_R^{2}=1-\frac{\left(1-R^{2})(n-1\right)}{n-p-1}$$

where *n* is the number of samples and *p* is the number of features.

## 3. Results and Analysis

### 3.1. Feature Extraction and Feature Selection from E-Nose System

**Figure 1:**
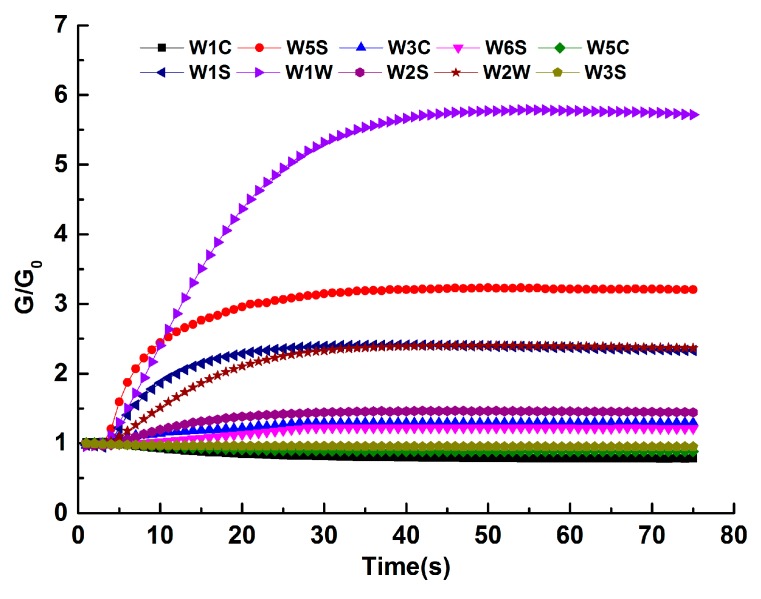
Sensor signal intensity of Jinjunmei tea.

**Figure 2:**
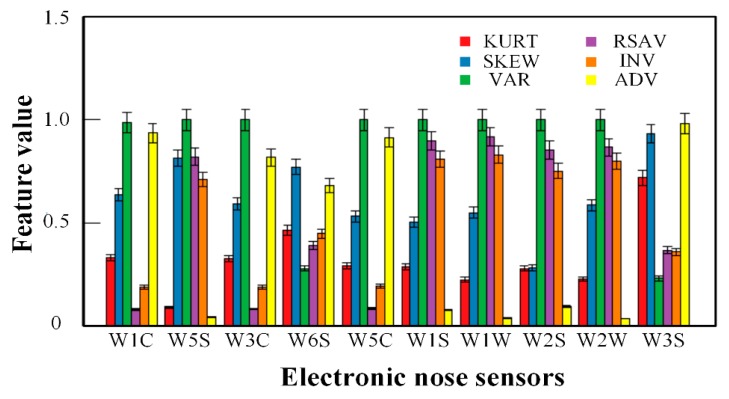
Bar results of six kinds of features of each gas sensor for one tea sample.

Figure 1 shows the response of the sensor array to Jinjunmei tea. As can be seen from Figure 2, the sensor W1W has a large response to the odor of the tea, and the response value is large. The sensors W5S, W1S, W2W, and W2S are positively deviated by 1, indicating that the response value is enhanced. The sensors W3C, W5C, and W1C deviate by 1 in the negative direction, indicating that the signal changes less. Therefore, different teas have different odors and the sensor signals obtained are different. It can be seen from Figure 1 that the response of the sensor tends to stabilize after 55 s. To facilitate data processing and correct differentiation, multiple features were extracted for each response curve.

Figure 2 shows a characteristic histogram of the response signal of each sensor for a tea sample. It can be seen from the figure that different characteristics reflect different response information for the same sensor, revealing that the gas sensor has broad spectrum responsiveness. However, the same feature also differs for different sensors, which reflects the selectivity of the sensor. Therefore, the sample data pattern generated by the array can be used to express the difference in tea quality and to achieve a one-to-one correspondence between the response pattern and the sample. Additionally, the array can be used to estimate the composition of a tea sample. For a single feature, the electronic nose signal consists of 10 features corresponding to 10 gas sensors. For six time domain features, the electronic nose signal is represented by 10 × 6 features. Therefore, the initial time domain feature matrix from the electronic nose is 110 × 60 features.

**Figure 3:**
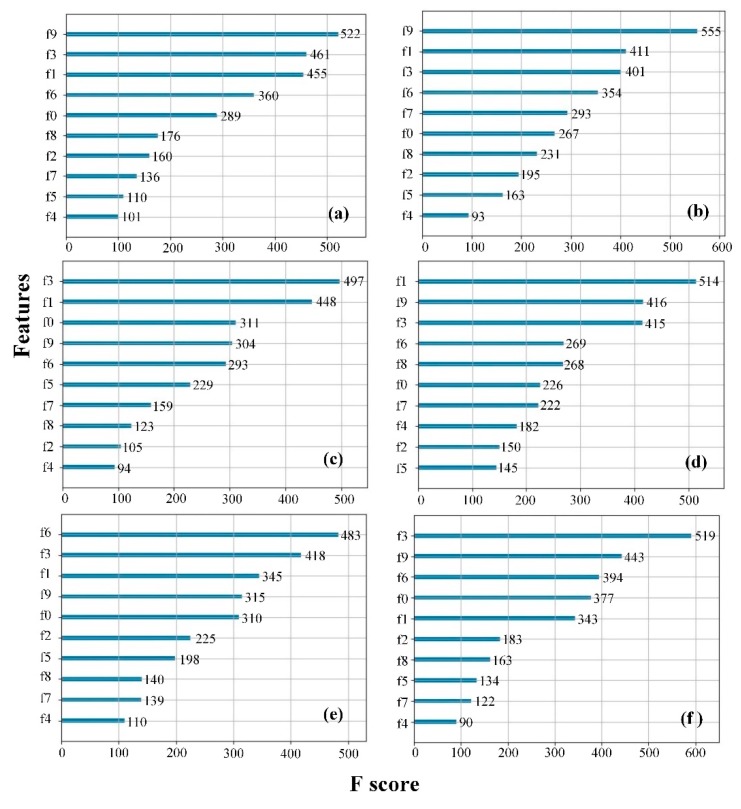
Feature importance: (**a**) variance value; (**b**) kurtosis coefficient; (**c**) integral value; (**d**) coefficient of skewness; (**e**) average differential value; (**f**) relative steady-state average value.

The feature importance based on XGBoost is shown in Figure 3, where the vertical axis represents features and the horizontal axis represents the number of times the feature is used to divide the decision tree nodes. As can be seen from Figure 3, the F-score of each feature for the preferred sensor array is different and the difference in performance is large. Therefore, this study ranks the top 30% of sensors according to the importance of the feature, and 3 × 6 features are preferred by XGBoost. For variance, kurtosis, and skewness, f1, f3, and f9 are selected according to importance; f0, f1, and f3 are selected for integral value (INV); f1, f3, and f6 are selected for average differential value (ADV); and f3, f6, and f9 are selected for relative steady-state average value (RSAV). The numbers of decision tree nodes for the above features are all above 400.

### 3.2. Feature Extraction and Feature Selection from HSI

**Figure 4:**
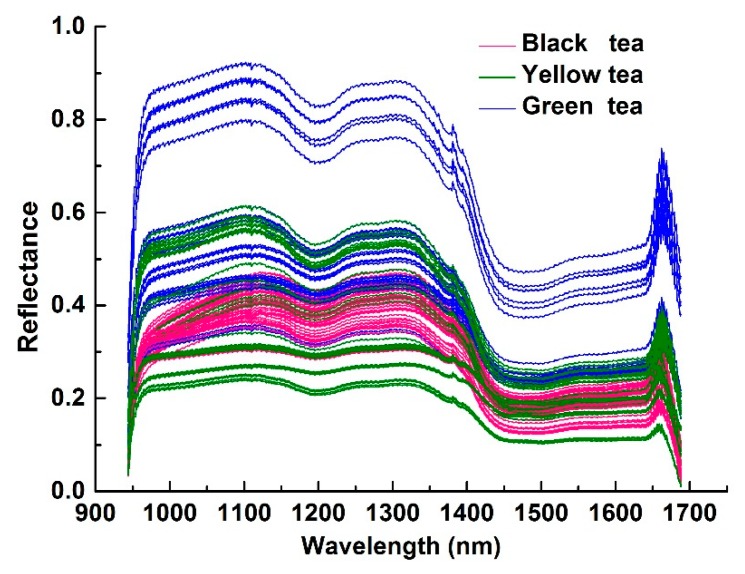
Near-infrared hyperspectral original curve of three categories of tea.

The 50 × 50 pixel area in the middle of the hyperspectral image was chosen as the region of interest (ROI). The spectral values of all the pixels of the ROI were extracted, and the average value was calculated as the spectral value of the sample. The spectral curves of the 110 tea samples are shown in Figure 4. There is significant noise at both ends of the spectrum. To improve the stability of the model, a total of 457 bands of 944–1688 nm were selected for further analysis. Successive projections algorithm (SPA) [40] was used to extract 1106 and 1375 nm as feature wavelengths for hyperspectral imaging. Time domain and frequency domain features were further extracted for hyperspectral images corresponding to feature wavelengths.

**Figure 5:**
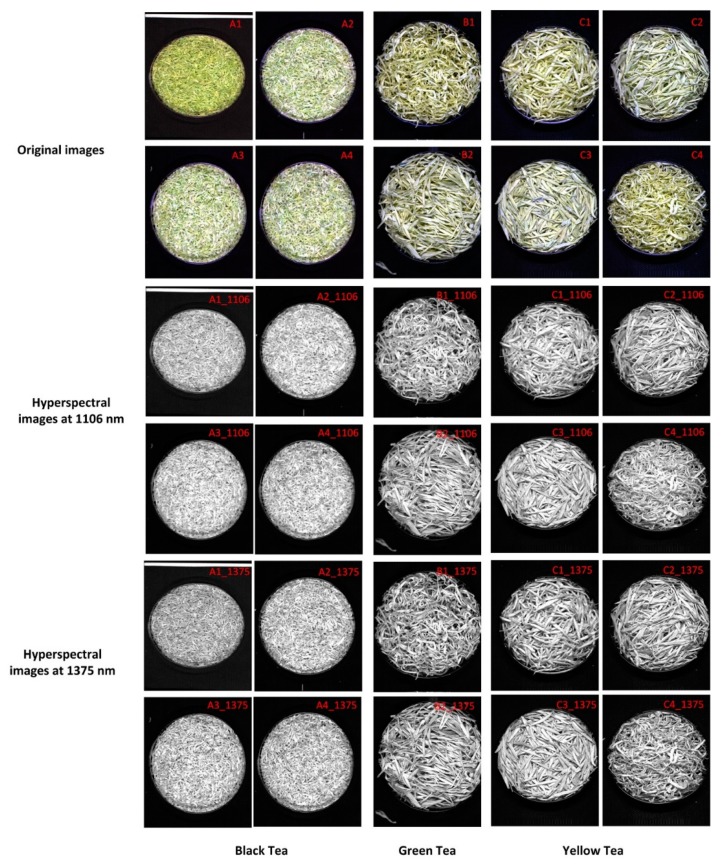
Original images and hyperspectral images based on characteristic wavelengths for three categories of tea. (A1): Zhengshan Xiaozhong ( Fujian); (A2): Qimen Black tea (Anhui Huangshan); (A3): Qimen Black tea ( Anhui Qimen); (A4): JinJunMei (Fujian); (B1): Huangshan Maofeng (Anhui); (B2): Liuan Guapian (Anhui); (C1): Junshan Yinzhen (Hunan); (C2): Huoshan Huangya tea (Anhui); (C3): Mengding Huangya tea (Sichuan) and (C4): Pingyang Huangtang tea (Zhejiang) represent the original images. A1_1106, A2_1106, A3_1106, A4_1106, B1_1106, B2_1106, C1_1106, C2_1106, C3_1106 and C4_1106 represent the 1106 nm hyperspectral images corresponding to the above original images; A1_1375, A2_ 1375, A3_1375, A4_ 1375, B1_1375, B2_1375, C1_1375, C2_1375, C3_1375 and C4_1375 represent the 1375 nm hyperspectral images corresponding to the above original images.)

Twenty-four features were extracted from hyperspectral images corresponding to characteristic wavelengths of tea samples (as shown in Figure 5), which describe the features of tea hyperspectral images for different feature parameters (entropy and energy), wavelet decomposition of different layers (two layers), and different directions (three directions). There are different correlations between the features and tea polyphenols.

**Figure 6:**
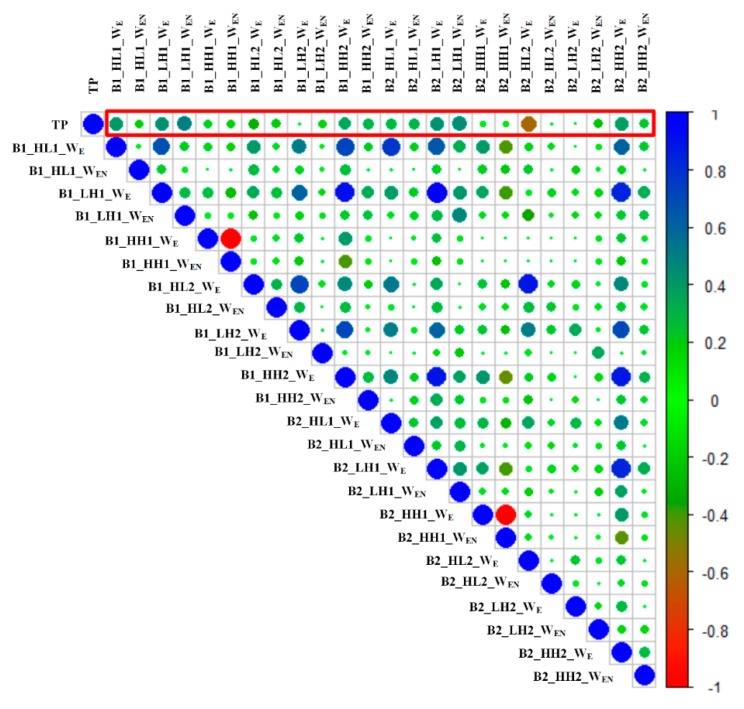
Correlation between tea polyphenols and spatial features of hyperspectral images.

In this study, Pearson’s correlation analysis was performed between 24 features and tea polyphenol content, as shown in Figure 6. The top 25% features with the absolute value of the correlation coefficient were selected. Therefore, six features that were positively correlated with TP were selected for further modeling, namely B1_HL1_WE, B1_LH1_WE, B1_LH1_WEN, B2_LH1_WE, and B2_LH1_WEN; along with B2_HL2_WE, which was negatively correlated with TP. The absolute values of the correlation coefficient of the above features ranged from 0.4 to 0.59.

### 3.3. Different Methods for Estimation of Polyphenol Content in Cross-Category Tea

The estimation models of tea polyphenol content were constructed with different features of cross-category tea, including time domain features and frequency domain features acquired from E-nose and HSI; and fusion features, which were compared with the model based on the original features, including the SVR, RF, and XGBoost models, as shown in Table 8. For the calibration set, R^2^ ranged from 0.839 to 0.977 for Grid-SVR, *R^2^* ranged from 0.758 to 0.876 for RF, and *R^2^* ranged from 0.992 to 0.998 for XGBoost. For the validation set, *R^2^* ranged from 0.703 to 0.816 for Grid-SVR, R^2^ ranged from 0.722 to 0.854 for RF, and *R^2^* ranged from 0.744 to 0.90 for XGBoost, which indicates that the estimation results based on models of Grid-SVR, RF, and XGBoost perform well.

### 3.4. Results of Models Based on Different Features

In the model constructed using all the original features, for calibration, the *R^2^* values of the model were 0.759–0.906 for E-nose, *R^2^* ranged from 0.758 to 0.995 for HSI, and *R^2^* was between 0.823 and 0.992 using the fusion features. For the validation set, *R^2^* values of the models were 0.703–0.808, 0.641–0.744, and 0.791–0.871 for E-nose, HSI, and fusion features, respectively.

**Figure 7:**
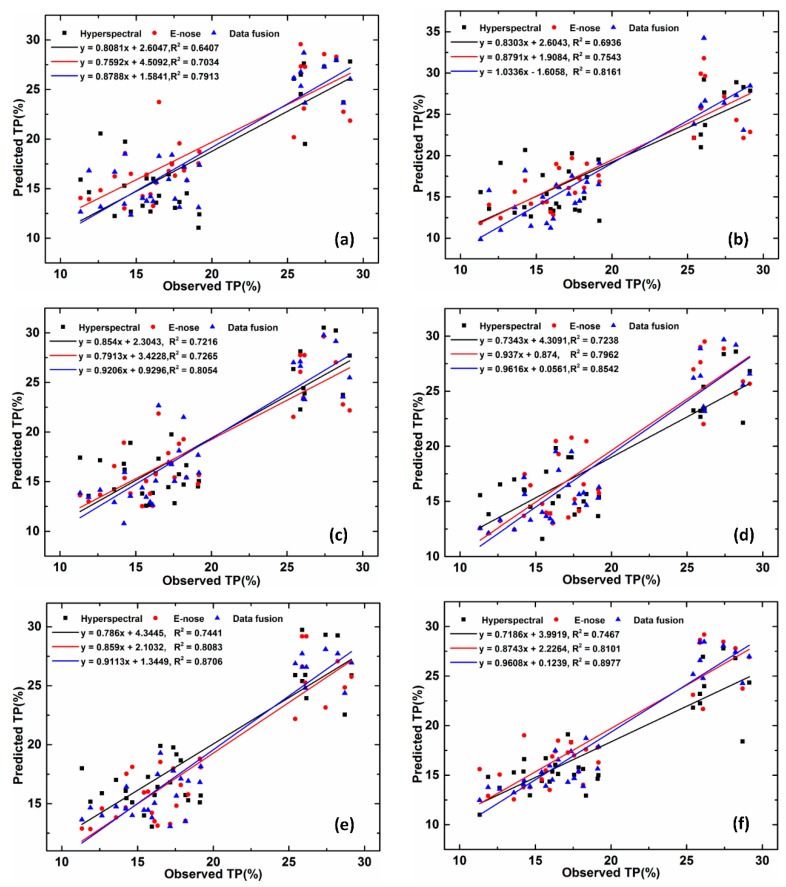
Estimation of polyphenol content of cross-category tea based on different features. Top: Grid-SVR model; middle: RF model; bottom: XGBoost model with original features (**a**,**c**,**e**) and preferred features (**b**,**d**,**f**).

All of the models were built using the preferred features from E-nose and HSI, including the Grid-SVR, RF, and XGBoost; these models performed better than those based on all features. As shown in Figure 7, especially for validation, *R^2^* for E-nose is between 0.754 and 0.81, *R^2^* for HSI is between 0.694 and 0.747, and *R^2^* for fusion features is between 0.816 and 0.90.

## 4. Discussion

### 4.1. Wavelet Transform and Features

Hyperspectral images with rich spectral information and image information contain numerous bands, which lead to excessive data dimensions. In addition, the spectral absorption features of tea polyphenols are mainly caused by the frequency doubling and frequency combination of basic chemical bonds, such as O–H and C–H in the molecules [58]. Therefore, in this study, the feature wavelengths of tea polyphenols were extracted according to the spectral curve, and the time domain features and frequency domain features were extracted according to the hyperspectral image corresponding to the feature wavelengths, which analyzes not only the spectral features, but also spatial features. From the spectral dimension, the hyperspectral images showed the performance of the material under different spectral signals. From spatial dimension analysis, an image is essentially a signal, which is a measure of the intensity variation between various locations of the image, including low frequency signals and high frequency signals. Moreover, the time domain and the frequency domain are the basic properties of the signal [59]. The time domain feature represents the time domain morphology and periodic variation of the signal, and the frequency domain analysis reflects the components of the signal. Therefore, the time domain features and frequency domain features extracted in this study are more representative.

Wavelet transform is the use of an orthogonal wavelet base to decompose the signal into components of different scales, which is equivalent to the partial decomposition of time series signals by using a set of high-pass and low-pass filters. Furthermore, the wavelet transform provides a “time frequency” window that varies with frequency, which obtains the detailed features of the signal by multiscale analysis of the signal stretching [45]. Therefore, regardless of the E-nose or HSI, the features extracted by wavelet transform are representative.

### 4.2. Different Features Affect Estimation Results

The number of features extracted will have a certain impact on the accuracy of the model [60]. Too few features cannot fully express the useful information of the data, which limits the accuracy of the model; too many features will increase the complexity and computation of the model. In order to obtain a good model estimation effect by applying the appropriate number of features, in this study, the feature selection methods were applied to the reduction of features from multisource sensors. Moreover, the cross-category tea polyphenol content prediction models constructed by feature fusion, such as Grid-SVR, RF, and XGBoost, showed that multisource feature fusion effectively improved the accuracy of the model.

Among them, the accuracy of the three models using the fusion features was higher than that of the individual features. For the Grid-SVR model, the *R^2^* values of the model based on the fusion features were 5.5% and 13.1% higher than the accuracy of the E-Nose and HSI features alone. The *R^2^* values of the RF model were increased by 3.1% and 12.6%, and the *R^2^* values of the XGBoost model were increased by 0.3% and 1.2%, respectively. Therefore, feature fusion can achieve effective data compression, facilitate real-time processing, and provide the information needed for decision analysis.

To verify the universality of the models, adjusted *R^2^* was used to re-evaluate the models, and the results are shown in Table 8. Regardless of whether using Grid-SVR, RF, or XGBoost models, the adjusted *R^2^* values of models based on the fusion features were the highest, which indicates that this is important for feature fusion to improve the accuracy of the model. At the same time, the adjusted R-square value as another evaluation index can eliminate the impact of the number of samples on the accuracy of the model, which further illustrates that the three models established are all valid.

### 4.3. Different Regression Models Affect Estimation Results

Table 8 shows that the estimated model established by the machine learning method in this paper performs well. Among them, the accuracy of the XGBoost model was higher than the Grid-SVR model or the RF model, which increased by 0.72% and 14.7% with the E-Nose feature, by 13.9% and 22.4% with the HSI feature, and by 2.1% and 12.2% with the fusion feature, respectively. The accuracy of the XGBoost estimation model, whether based on a single feature or a fused feature, was high. In fact, all three methods have their own characteristics. XGBoost and RF are integrated learning methods based on boosting and bagging, respectively. Grid-SVR is an SVR model whose parameters are optimized by grids, and which performs well.

In addition, the methods based on fusion features obtained from E-nose and HSI can provide different methods for predicting the tea polyphenol content of cross-category tea. Wang et al. estimated the polyphenol content of five varieties of green tea [61]. Wang et al. estimated polyphenol contents four Chinese tea, including black tea, dark tea, oolong, and green tea [62]. Although they all achieved good results, they were all based on NIR (near-infrared reflectance) spectroscopy, lacking comprehensive feature fusion. However, the time domain features and frequency domain features from different sensors in this study can provide a new approach for estimating tea composition. The proposed method was used to obtain the expected predictions, despite dealing with different categories, different brand samples, different sensors, and different features. In the future, more varieties of tea should be collected to further improve the universality of the model and provide technical support for nondestructive testing of tea.

## 5. Conclusions

In this study, an estimation model for polyphenol content in cross-category tea based on feature fusion was proposed, including time and frequency domains, and cross-category tea varieties, such as black tea, green tea, and yellow tea. On the one hand, fusion-based models are superior to models based solely on electronic nose or hyperspectral features, as multisensor feature fusion provides more features, including time and frequency domain features, which reflect gas-sensitive information, spectral and spatial information. The Grid-SVR, RF, and XGBoost models were constructed to estimate the polyphenol content of cross-category teas, with the XGBoost model performing best out of the three models (*R^2^* = 0.998 and adjusted *R^2^* = 0.995 for calibration, *R^2^* = 0.90 and adjusted *R^2^* = 0.75 for validation), which provides a technical basis for quantitative estimation of tea polyphenol content of cross-category tea. In addition, the method proposed in this paper can be used to nondestructively detect the components of other varieties of tea, such as the amino acids and tea polyphenols of white tea, dark tea, and Pu’er tea in future research.

## Figures and Tables

**Table 1:** Geographical sources and descriptive statistics of tea polyphenol content (%).

Tea Category	Tea Variety (Geographical Origins)	Number	Range (%)	Mean ± SD (%)
Black tea	Zhengshan Xiaozhong (Fujian)	10	10.65–13.21	11.832 ± 0.850
	Qimen Black Tea (Anhui Huangshan)	10	13.66–16.54	15.196 ± 0.810
	Qimen Black Tea (Anhui Qimen)	10	16.51–22.85	18.789 ± 1.567
	JinJunMei (Fujian)	10	12.62–19.16	16.99 ± 1.788
Green tea	Huangshan Maofeng (Anhui)	15	25.32–29.41	26.485 ± 1.195
	Liuan Guapian (Anhui)	15	27.42–29.65	28.535 ± 0.632
Yellow tea	Junshan Yinzhen (Hunan)	10	14.55–19.6	16.34 ± 1.863
	Huoshan Huangya Tea (Anhui)	10	11.88–16.65	13.862 ± 1.367
	Mengding Huangya Tea (Sichuan)	10	11.36–16.35	14.334 ± 1.738
	Pingyang Huangtang Tea (Zhejiang)	10	13.34–19.36	16.735 ± 2.231

**Table 2:** Performance description of sensors for the PEN3 electronic nose (E-nose).

Array Number	Sensor Name	Object Substances of Sensing	Component	Threshold Value/(mL m^−3^)
f0	W1C	Aromatics	C_6_H_5_CH_3_	10
f1	W5S	Nitrogen oxides	NO_2_	1
f2	W3C	Ammonia and aromatic molecules	C_6_H_6_	10
f3	W6S	Hydrogen	H_2_	100
f4	W5C	Methane, propane and aliphatic nonpolar molecules	C_3_H_8_	1
f5	W1S	Broad methane	CH_4_	100
f6	W1W	Sulfur-containing organics	H_2_S	1
f7	W2S	Broad alcohols	CO	100
f8	W2W	Aromatics, sulfur-, and chlorine-containing organics	H_2_S	1
f9	W3S	Methane and aliphatics	CH_4_	10

**Table 3:** Feature extraction from electronic nose signals.

Indices	Name	Formula
VAR	Variance value	$VAR=\frac{1}{N}\sum_{i=1}^{N}{\left(c_{i}-\bar{c}\right)}^{2}$
INV	Integral value	INV=c(i)Δt
RSAV	Relative steady-state average value	$RSAV=\frac{1}{N-t_{0}}\sum_{t_{o}}^{T}c_{{}^{i}}$
ADV	Average differential value	$ADV=\frac{1}{N-1}\sum_{i=1}^{N-1}\frac{c_{i+1}-c_{i}}{\Delta t}$
KURT	Kurtosis coefficient	$KURT=\frac{\frac{1}{N}\sum_{i=1}^{N}{\left(c_{i}-\bar{c}\right)}^{4}}{(\frac{1}{N}\sum_{i=1}^{N}{\left({\left(c_{i}-\bar{c}\right)}^{2}\right)}^{2}}-3=\frac{m_{{}^{4}}}{m_{2}^{2}}-3$
SKEW	Coefficient of skewness	$SKEW=\frac{\sqrt{N(N-1)}}{N-2}\frac{\frac{1}{N}\sum_{i=1}^{N}{\left(c_{i}-\bar{c}\right)}^{3}}{(\frac{1}{N}\sum_{i=1}^{N}{\left({\left(c_{i}-\bar{c}\right)}^{2}\right)}^{\frac{3}{2}}}$

**Table 4:** The support vector regression (SVR) modeling hyperparameters.

Parameter	Range	Optimum Value
c	0 to 20	15
g	0 to 10	5
s	0 to 10	3
p	0.001 to 1	0.01

**Table 5:** The random forest (RF) regression modeling hyperparameters.

Parameter	Range	Optimum Value
n_estimators	100 to 2000	1000
max_depth	1 to 10	3
extra_ options. importance	[0, 1]	1
extra_ options. nPerm	[0, 1]	1
bootstrap	[True, False]	FALSE

**Table 6:** Extreme gradient boosting (XGBoost) regression modeling hyperparameters.

Parameter	Range	Optimum Value
learning_rate	0.1 to 1	0.1
n_estimators	100 to 1000	400
max_depth	1 to 10	5
gamma	0.1 to 1	0.1
subsample	0.1 to 1	0.9
min_child_weight	3 to 10	5

**Table 7:** Descriptive statistics of tea polyphenol (TP) contents in the calibration and prediction sets.

Data Set	Number	Content Range	Mean	SD
Full	110	10.65–29.65	18.78	5.82
Calibration set	80	10.65–29.65	18.61	5.94
Validation set	30	11.31–29.13	19.21	5.47

**Table 8:** Estimation models for polyphenol content of cross-category tea based on different variables with three techniques. Note: Grid-SVR = grid support vector regression; RF = random forest; XGBoost = extreme gradient boosting; RMSE = root mean square error.

Model	Features	Variables	Calibration			Validation		
		Number	*R^2^*	*Adjusted_R^2^*	RMSE	*R^2^*	*Adjusted_ R^2^*	RMSE
**Grid-SVR**	E-Nose	20	0.923	0.819	1.659	0.754	0.472	2.852
	HSI	6	0.849	0.705	2.313	0.694	0.451	3.225
	Fusion	26	0.977	0.940	0.906	0.816	0.561	2.856
RF	E-Nose	20	0.848	0.656	2.318	0.796	0.551	2.637
	HSI	6	0.765	0.561	2.881	0.724	0.496	2.982
	Fusion	26	0.876	0.695	2.094	0.854	0.645	2.287
XGBoost	E-Nose	20	0.995	0.988	0.274	0.810	0.579	2.422
	HSI	6	0.987	0.973	0.705	0.747	0.532	3.099
	Fusion	26	0.998	0.995	0.434	0.900	0.750	1.895
